# Biochemical and structural characterization of a thermostable Dps protein with His‐type ferroxidase centers and outer metal‐binding sites

**DOI:** 10.1002/2211-5463.12837

**Published:** 2020-05-28

**Authors:** Takuo Minato, Takamasa Teramoto, Yoshimitsu Kakuta, Seiji Ogo, Ki‐Seok Yoon

**Affiliations:** ^1^ Department of Chemistry and Biochemistry Graduate School of Engineering Kyushu University Fukuoka Japan; ^2^ International Institute for Carbon‐Neutral Energy Research (WPI‐I2CNER) Kyushu University Fukuoka Japan; ^3^ Department of Bioscience and Biotechnology Faculty of Agriculture Kyushu University Fukuoka Japan; ^4^ Laboratory of Structural Biology Graduate School of System Life Sciences Kyushu University Fukuoka Japan; ^5^ Center for Small Molecule Energy Kyushu University Fukuoka Japan

**Keywords:** cyanobacteria, DNA‐binding protein from starved cells, His‐type ferroxidase center, metal‐binding site, thermostability

## Abstract

The DNA‐binding protein from starved cells (Dps) is found in a wide range of microorganisms, and it has been well characterized. However, little is known about Dps proteins from nonheterocystous filamentous cyanobacteria. In this study, a Dps protein from the thermophilic nonheterocystous filamentous cyanobacterium *Thermoleptolyngbya* sp. O‐77 (*Tl*Dps1) was purified and characterized. PAGE and CD analyses of *Tl*Dps1 demonstrated that it had higher thermostability than previously reported Dps proteins. X‐ray crystallographic analysis revealed that *Tl*Dps1 possessed His‐type ferroxidase centers within the cavity and unique metal‐binding sites located on the surface of the protein, which presumably contributed to its exceedingly high thermostability.

## Graphical abstract

A DNA‐binding protein from starved cells (Dps) protein from nonheterocystous filamentous cyanobacterium *Thermoleptolyngbya* sp. O‐77 (*Tl*Dps1) was purified and characterized. PAGE and CD analyses of *Tl*Dps1 illustrated that it had higher thermostability than previously reported Dps proteins. X‐ray crystallographic analysis revealed that *Tl*Dps1 possessed His‐type ferroxidase centers within the cavity and unique metal‐binding sites located on the surface of the protein.

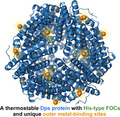

**Abbreviations**
Dps: DNA‐binding protein from starved cells
FOC: ferroxidase center
ICP: inductively coupled plasma
ISD: in‐source decay
LMCT: ligand‐to‐metal charge transfer
MALDI‐TOF: matrix‐assisted laser desorption/ionization time‐of‐flight
OMS: outer metal‐binding site
ROS: reactive oxygen species
SEC‐RALS: size exclusion chromatography coupled to right angle light scattering

The DNA‐binding protein from starved cells (Dps) is distributed widely in the bacterial kingdom, and it belongs to the ferritin protein family [1]. Ferritin consists of 24 subunits with the point group symmetry of *O*, whereas Dps consists of 12 subunits with the point group symmetry of *T*, resulting in one of the smallest spherical protein cages [2, 3]. Dps proteins can store Fe cations as oxyhydroxide clusters within its cavity similarly as ferritin, partially explaining its role in iron homeostasis [4]. The distinctive property of Dps proteins allows them to serve as defense systems against reactive oxygen species (ROS) using ferroxidase centers (FOCs) located at the interfaces between subunits. FOCs catalyze the oxidative conversion of Fe^2+^ to Fe^3+^ using O_2_ and/or H_2_O_2_ to protect DNA from oxidative damage caused by the Fenton reaction, and Dps proteins take up Fe cations within the cavity through pores [5]. Because FOCs have a crucial role in cell survival under oxidative stresses, the DNA‐binding and iron uptake ability of Dps proteins together with crystal structures of FOCs from various types of bacteria have been revealed [3, 6, 7, 8, 9, 10, 11, 12, 13, 14, 15, 16, 17, 18, 19, 20, 21, 22, 23, 24]. Conventional FOCs in Dps proteins consist of highly conserved amino acids, including two His, one Trp, one Asp, and one Glu residue, and two Fe cations are coordinated to these amino acid residues. Recently, a new type of FOCs in DpsA from the cyanobacterium *Thermosynechococcus elongatus* BP‐1 (*Te*DpsA) has been reported. In this protein, the FOCs consist of four His and one Glu residue with Zn cations [6]. This new type of FOC was named the His‐type FOC in a study of the Dps4 protein from the cyanobacterium *Nostoc punctiforme* ATCC 29133 (*Np*Dps4) [7]. In this report, the genomic database illustrated that the His‐type FOC‐containing Dps protein is widely found only in cyanobacteria, thus indicating that His‐type FOCs may be unique to cyanobacterial Dps proteins. However, because there have been only two reports of His‐type FOC‐containing Dps proteins from unicellular and filamentous cyanobacteria including *Te*DpsA and *Np*Dps4, the nature of this new class of proteins from other cyanobacteria remains unknown.

To investigate Dps proteins from previously unstudied filamentous nonheterocystous cyanobacteria, *Thermoleptolyngbya* sp. O‐77 (*Tl*. O‐77) isolated from a hot spring in Japan was utilized as a model. *Tl.* O‐77 grows well over a temperature range of 308–333 K and exhibits thermophilic behavior with an optimal growth temperature of 328 K in nitrate‐containing medium under aerobic conditions. Previously, an oxygen‐evolving photosystem II (PSII) from *Tl.* O‐77 was purified, and its significant thermotolerance as compared with other PSII from cyanobacteria and higher plants was revealed [25]. According to its genome, *Tl.* O‐77 possesses two types of His‐type FOC‐containing Dps‐encoding genes, indicating that the expressed Dps proteins are expected to display high thermostabilities [26]. We envisaged that investigation of the His‐type FOC‐containing Dps proteins from *Tl.* O‐77 would unveil the nature of unexplored Dps proteins from thermophilic filamentous bacteria and expand knowledge about His‐type FOC systematically. In this study, a Dps protein from *Tl.* O‐77 (*Tl*Dps1) was successfully purified, and its native state was characterized. X‐ray crystallographic analysis revealed that *Tl*Dps1 possessed His‐type FOCs together with unique metal‐binding sites located on the surface of the protein. Moreover, *Tl*Dps1 exhibited higher thermostability than all previously reported Dps proteins.

## Materials and methods

### Growth condition of *Tl.* O‐77

Cells were grown in a 10‐L clear glass bottles containing a modified DH + Fe medium at 315 K under a constant flow (200 mL·min^−1^) of air, and the cultures were continuously illuminated with 60 W incandescent lamps at 60 µmol m^−2^·s^−1^. The modified DH + Fe medium was prepared with the following components (per liter): N(CH_2_COOH)_3_ (100 mg), NaNO_3_ (1.70 g), K_2_HPO_4_ (140 mg), MgSO_4_·7H_2_O (100 mg), CaSO_4_·2H_2_O (100 mg), FeCl_3_ (30.0 mg), and the trace metal solution (1.0 mL), where the trace metal solution (1.0 L) contains H_3_BO_3_ (3.00 g), MnSO_4_·H_2_O (2.00 g), ZnSO_4_ (50.0 mg), CoCl_2_·6H_2_O (37.5 mg), NiCl_2_·6H_2_O (37.5 mg), and CuSO_4_ (40.0 mg). The pH of the medium was adjusted to 7.5 by addition of NaOH.

### Purification of *Tl*Dps1

The harvested cells (wet weight, 150 g) were homogenized with 800 mL of 20 mm Tris/HCl buffer at pH 8.0 (buffer A) using NZ‐1000 (EYELA, Tokyo, Japan) and disrupted in an ice bath by sonication three times (2‐min sonication at 30 W and 2‐min break) using an Ultrasonic Disruptor UD‐200 (TOMY SEIKO, Tokyo, Japan). Cell debris and unbroken cells were removed by centrifugation (5000 ***g***, 20 min, 277 K) using himac CR20GII (HITACHI, Tokyo, Japan), and the resulting supernatant was centrifuged at 100 000 ***g*** for 1 h using an Optima L‐90K (Beckman Coulter, Brea, CA, USA). Then, the resulting supernatant of soluble cell extracts containing 0.15 m NaCl was loaded onto a DEAE Sepharose fast flow column (XK 50/20; GE Healthcare Life Sciences, Buckinghamshire, UK) pre‐equilibrated with the buffer A by washing with 500 mL of the same buffer at the flow rate of 10 mL·min^−1^. The *Tl*Dps1‐containing solution was eluted at 0.3–0.4 m NaCl with 22–28 mS·cm^−1^ ionic conductivity by using the buffer A and the buffer A containing 1.0 m NaCl (buffer B) as eluents. The fractions containing *Tl*Dps1 were combined and diluted threefold with the buffer A. The resulting solution was loaded onto a Q Sepharose high‐performance column (HR 16/10; GE Healthcare Life Sciences) pre‐equilibrated with the buffer A by washing with 150 mL of the same buffer at a flow rate of 5 mL·min^−1^. The pale yellow *Tl*Dps1‐containing solution was eluted at 0.36 m NaCl with 31–32 mS·cm^−1^ ionic conductivity by using the buffers A and B. The fractions containing *Tl*Dps1 were combined and diluted twofold with 2.0 m (NH_4_)_2_SO_4_ aqueous solution. The resulting solution was loaded onto a Phenyl Sepharose high‐performance column (HR 16/10; GE Healthcare Life Sciences) pre‐equilibrated with the buffer A containing 1.0 m (NH_4_)_2_SO_4_ (buffer C). The column was washed with 150 mL of the same buffer at a flow rate of 5 mL·min^−1^. The pale yellow *Tl*Dps1‐containing solution was eluted at 0.2–0.4 m (NH_4_)_2_SO_4_ with 20–65 mS·cm^−1^ ionic conductivity by using the buffers A and C. The fractions containing *Tl*Dps1 were combined and concentrated using Amicon Ultra‐15 30 kDa (Merck, Darmstadt, Germany). The resulting concentrated *Tl*Dps1 was purified using Superdex 200 prep grade column (HR 16/50; GE Healthcare Life Sciences). The concentration of the purified *Tl*Dps1 solution was determined by the Lowry method using DC protein assay (Bio‐Rad, Hercules, CA, USA). Final amount of *Tl*Dps1 obtained from the harvested cells (wet weight, 150 g) was ca. 1.5 mg.

The apo form of *Tl*Dps1 (*Tl*apoDps1) was prepared as follows: Aqueous solution of sodium hydrosulfite (14.5 mg, 500 µL) was added to the *Tl*Dps1 solution (4.0 mg·mL^−1^, 200 µL) of 10 mm Tris/HCl buffer at pH 8.0 under nitrogen gas. The resulting solution was incubated at room temperature for 24 h followed by the concentration using Amicon Ultra‐15 30 kDa (Merck). Then, the concentrated *Tl*apoDps1 was purified using Sephadex G‐25 column (HR 16/10; GE Healthcare Life Sciences) with 10 mm Tris/HCl buffer at pH 8.0 to separate *Tl*apoDps1 and other small molecules, such as Fe^2+^ and unreacted sodium hydrosulfite. Fractions containing *Tl*apoDps1 were collected, and the same procedure was repeated twice to give a colorless *Tl*apoDps1 solution.

### Polyacrylamide gel electrophoresis (PAGE) analysis

Native PAGE and SDS/PAGE were performed by WSE‐1150 PageRunAce (ATTO, Tokyo, Japan) using ET520L 5–20% gel (ATTO). The sample buffer for SDS/PAGE contains 60 mm Tris/HCl buffer at pH 6.8, 25% glycerol, 2% SDS, 14.4 mm 2‐mercaptoethanol, and 0.1% bromophenol blue. DNase‐treated *Tl*Dps1 was prepared as follows: 2 µL of RNase‐free DNase I (NIPPON GENE, Tokyo, Japan) was added to the mixed solution of a *Tl*Dps1 solution (141 µg·mL^−1^, 88 µL) containing 10 mm Tris/HCl buffer at pH 8.0 and a buffer solution (10 µL) containing 0.4 m Tris/HCl buffer at pH 7.9, 0.1 m NaCl, 60 mm MgCl_2_, and 10 mm CaCl_2_. The resulting solution was incubated at 303 K for 30 min before loading onto the gel.

### Spectral analysis

Matrix‐assisted laser desorption/ionization time‐of‐flight (MALDI‐TOF) mass spectrometric analysis of *Tl*Dps1 protein was measured using a microflex LT (Bruker Daltonics, Billerica, MA, USA). The sample was prepared by mixing the desaltified aqueous solution of *Tl*Dps1 (0.5 µL) with a saturated solution of 1,5‐naphthalenediamine (1 µL) in a mixed solvent of acetonitrile and 0.1% trifluoroacetic acid (1 : 1, v/v). The resulting sample was deposited onto a ground steel MALDI target plate and dried at room temperature (ca. 298 K). In‐source decay (ISD) fragments of the sample were measured at the laser intensity of 50% for 35 000 times. External mass calibration was performed using [M + H]^+^ ions of the protein standard II (Bruker Daltonics).

Metal contents of *Tl*Dps1 were analyzed by the inductively coupled plasma (ICP) mass measurement using an Agilent 7500 (Agilent, Santa Clara, CA. USA) with H_2_ mode. The numbers of metal cations per dodecamer were calculated using the obtained concentrations of metal cations, mass of iron oxyhydroxide (88.85), and mass of dodecameric form of *Tl*Dps1 without metal cations (247790.88) based on a calibration‐curve method. The sample concentrations were 82.4 ppb (*Tl*Dps1 for Fe), 824.0 ppb (*Tl*Dps1 for Zn), and 840.0 ppb (*Tl*apoDps1 for Zn and Fe).

CD measurement was performed using Chirascan (Applied Photophysics, Skipton, UK) with a 0.1‐cm quartz cell. Temperature dependences of CD were measured at the temperature ramp rate of 1.0 K·min^−1^ with 0.5 K step in a temperature range of 293–368 K. Time‐course CD spectra at 222 nm were measured at 368 K.

UV–vis spectra were measured on JASCO V‐670 with 1‐cm quartz cells. The concentrations of *Tl*Dps1 and *Tl*apoDps1 were 0.1 mg·mL^−1^.

Size exclusion chromatography coupled to right angle light scattering (SEC‐RALS) was performed using Viscotek TDAmax (Malvern Panalytical, Almelo, Netherlands) and Superdex 200 Increase column (GE Healthcare Life Sciences) at 303 K with 10 mm Tris/HCl buffer at pH 8.0 at 0.5 mL·min^−1^. Concentrations of the samples, *Tl*Dps1 and *Tl*apoDps1, were 206 and 168 µg·mL^−1^, respectively.

### Crystallization and data collection

Crystallization was performed at 293 K by the hanging drop vapor diffusion technique. A *Tl*Dps1 solution (4.0 mg·mL^−1^, 2 μL) in 10 mm Tris/HCl buffer (pH 8.0) was mixed with the reservoir solution (1 µL) containing 0.15 m cesium chloride and 18% polyethylene glycol 3350. Crystals grew in 3 days to 0.1 × 0.1 × 0.1 mm. The crystal was flash‐frozen in liquid nitrogen. A 2.9‐Å‐resolution data set was collected at 1.25 Å at beamline BL45XU, SPring‐8, Hyogo, Japan. The anomalous X‐ray diffraction data with different wavelengths (1.25 and 1.32 Å) were also collected to determine the position of Zn because anomalous signals of Zn *K* edge were observed at 1.25 Å and were hardly observed at 1.32 Å considering the X‐ray Zn absorption edge of 1.28 Å. The data were processed using ZOO system and XDS [27, 28, 29].

### Structure determination and refinement

The structure was determined by molecular replacement using the program Phaser and search models of one subunit of *Np*Dps4 (PDB ID: 5HJH) [30]. Model building was carried out with the program coot [31]. The program Phenix.refine was used for refinement [32]. The structure displayed good geometry when analyzed by MolProbity [33]. 95.44% and 4.56% of the residues constituting *Tl*Dps1 were in the most favored and allowed regions of the Ramachandran plot, respectively.

## Results and Discussion

### Identification of *Tl*Dps1

**Fig. 1:**
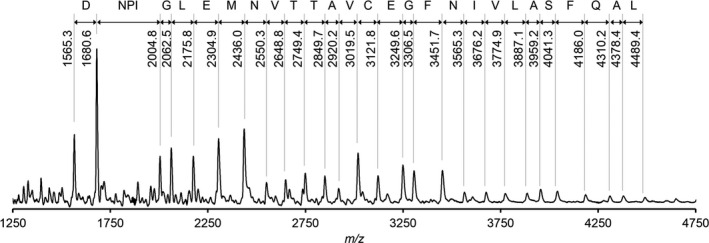
ISD‐MALDI‐TOF mass spectrum of *Tl*Dps1. N‐terminal fragment ions from the 16th to 43rd provided the amino acid sequence for a continuous stretch of 28 residues in *Tl*Dps1.

The recently isolated *Tl.* O‐77 was used as a model to study Dps proteins from thermophilic nonheterocystous filamentous cyanobacteria. Based on the genome sequence analysis, *Tl.* O‐77 possesses four distinct Dps‐encoding genes (*Tl*Dps1–4). ISD‐MALDI‐TOF mass measurement of the isolated Dps protein revealed 27 major signals assignable to the N‐terminal fragments of *Tl*Dps1 that corresponded to the amino acid sequence for a continuous stretch of DNPIGLEMNVTTAVCEGFNIVLASFQAL (Fig. 1), indicating that the isolated Dps protein was *Tl*Dps1 (Fig. S1). The mass spectrum also revealed a signal at *m/z* 1565.6 assignable to the protonated N‐terminal fragment of AITASPIQTFEQMK (*m/z* 1564.8), suggesting that the N terminus (Met1 in *Tl*Dps1) was deleted via a post‐translational modification.

Compared with the sequences of conventional Dps proteins, *Tl*Dps1 possesses unique amino acids including Phe52, His78, and His164, which are characteristic of Dps proteins with His‐type FOCs as previously isolated from the cyanobacteria *T. elongatus* and *N. punctiforme* [6, 7]. A sequence similarity search illustrated that *Tl*Dps1 shares 66% and 70% sequence identity with the His‐type FOC‐containing proteins *Te*DpsA and *Np*Dps4, respectively, whereas it shares low sequence identities with conventional Dps proteins from bacteria such as *Escherichia coli* (*Ec*Dps, 27%), *Listeria innocua* (*Li*Dps, 27%), *Mycobacterium smegmatis* (*Ms*Dps1, 28%), *Deinococcus radiodurans* (*Dr*Dps1, 28%), and *T. elongatus* (*Te*Dps, 30%). Although an archaeal Dps protein from the halophilic archaeon *Halobacterium salinarum* (*Hs*DpsA) possesses His165 as a metal‐binding site in its FOC [8], the main components of the FOC are identical to those in conventional Dps proteins (Fig. S1). Interestingly, *Tl*. O‐77 possesses two distinct His‐type FOC‐containing Dps‐encoding genes, namely *Tl*Dps1 and *Tl*Dps2, whereas *T. elongatus* and *N. punctiforme* each possess one His‐type FOC‐containing Dps‐encoding gene (*Te*DpsA and *Np*Dps4, respectively). Cyanobacteria commonly carry multiple Dps‐encoding genes; however, there have been no reports of bacteria that possess multiple His‐type FOC‐containing Dps‐encoding genes.

### Overall crystal structure of *Tl*Dps1

**Fig. 2:**
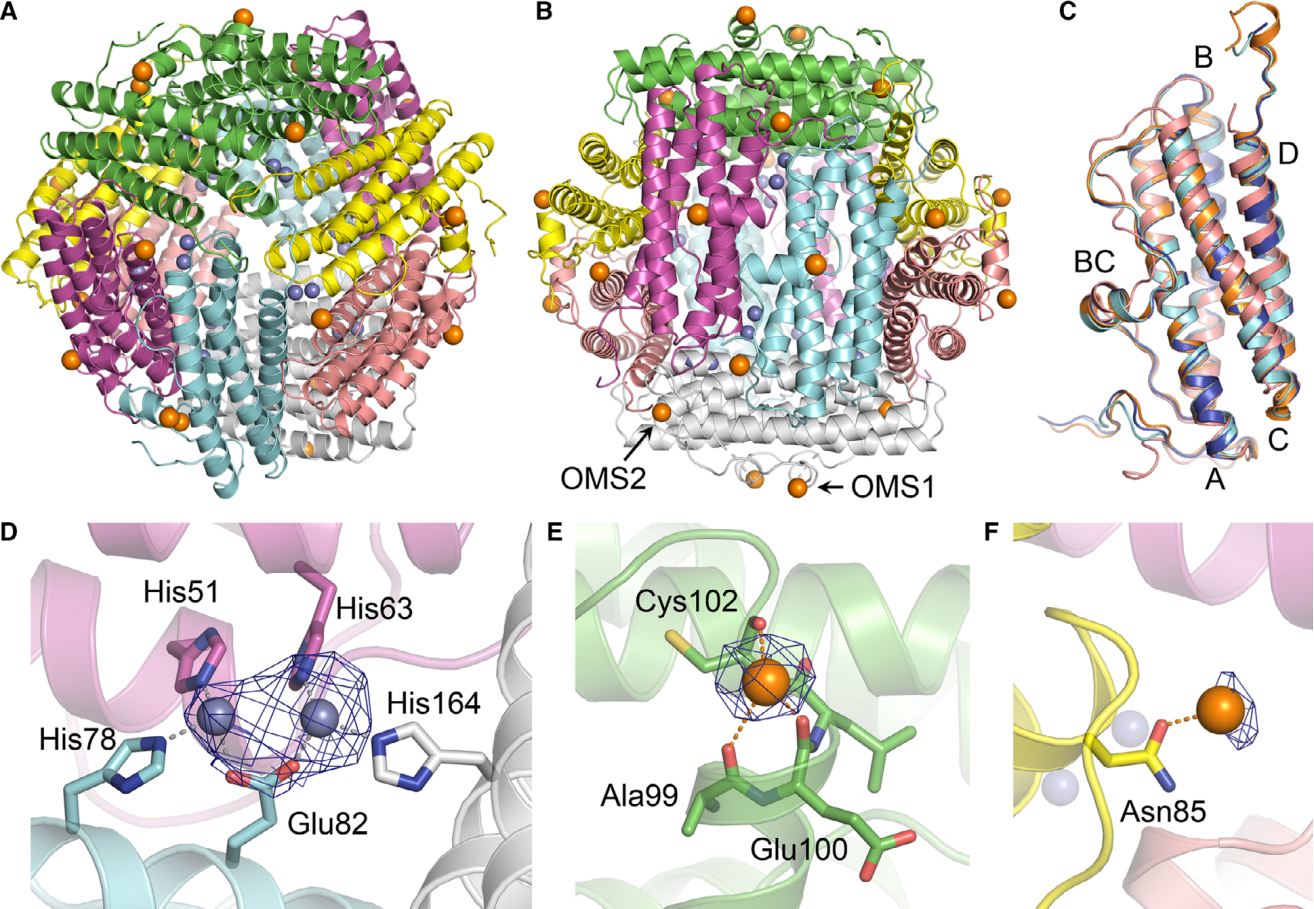
Cartoon representations of *Tl*Dps1 along the (A) *C*_3_ and (B) *C*_2_ symmetry axes based on the X‐ray crystallographic analysis. (C) Superposition of Dps monomers for *Tl*Dps1 (pale blue), *Ec*Dps (pink; PDB ID, 1DPS), *Te*DpsA (blue; PDB ID, 2VXX), and *Np*Dps4 (orange; PDB ID, 5HJF). Capital letters represent corresponding helix bundles (A, B, BC, C, and D). Cartoon representations of (D) His‐type FOC, (E) OMS1, and (F) OMS2 in *Tl*Dps1. *F*_o_–*F*_c_ omit electron density maps contoured at 3.0σ are superimposed on metal cations. Zn and Fe atoms are represented as gray and orange spheres, respectively.

The brown single crystals of *Tl*Dps1 were obtained using the hanging drop method (Fig. S2). X‐ray crystallographic analysis revealed that *Tl*Dps1 consists of 12 subunits (six subunits in an asymmetric unit) that form a spherical cage‐like structure with a diameter of ~ 9 nm (Table S1 and Fig. 2A,B). The negatively charged amino acid residues Glu64, Glu68, Asp71, Glu72, Glu79, Glu82, Glu157, and Asp160 are located on the inner surface of the cavity with a diameter of ~ 4 nm, which enables *Tl*Dps1 to store Fe cations as iron oxyhydroxide clusters. The UV–vis spectrum of *Tl*Dps1 in 10 mm Tris/HCl buffer at pH 8.0 revealed absorption bands at ~ 350 nm that were assignable to the O^2–^‐to‐Fe^3+^ ligand‐to‐metal charge transfer (LMCT) process (Fig. S3), supporting the presence of iron oxyhydroxide clusters in the cavity of *Tl*Dps1. The number of Fe cations as FeOOH was estimated as ~ 420 per dodecamer via ICP mass measurement, which was in good agreement with the absolute molecular weight (289 675) obtained via SEC‐RALS analysis. The monomers of *Tl*Dps1 folded into four α‐helix bundles stabilized mainly by hydrophobic interactions, which is a typical tertiary structure for Dps proteins (Fig. 2C). The CD spectrum of *Tl*Dps1 revealed negative Cotton effects with maxima at 222 and 208 nm and a positive Cotton effect with a maximum at 195 nm, which are characteristic bands of α‐helix structures, reflecting the crystal structure of *Tl*Dps1 (Fig. S4). Compared with the findings in Dps proteins including *Ec*Dps, *Te*DpsA, and *Np*Dps4, the His‐type FOC‐containing Dps proteins (*Te*DpsA, *Np*Dps4, and *Tl*Dps1) exhibit similar tertiary structures (Fig. 2C).

Within the cavity, 12 metal‐binding sites of FOCs were formed in interfaces between subunits (Fig. 2D). The FOCs of *Tl*Dps1 consisted of five amino acid residues, namely His51, His63, His78, Glu82, and His164, which are typical for His‐type FOCs. As the metal cations coordinated to the His‐type FOCs of *Te*DpsA and *Np*Dps4 were assigned to Zn, the types of metal cations in the FOCs of *Tl*Dps1 were deduced to be Zn. Considering the X‐ray Zn absorption edge of 1.28 Å, the anomalous X‐ray diffraction measurements with different wavelengths were performed. The anomalous electron density map obtained from data collected at an energy above the Zn *K* edge (1.25 Å) showed anomalous signals assignable to Zn, whereas that obtained from data collected at an energy below the Zn K edge (1.32 Å) hardly showed anomalous signals, thus clearly illustrating the type of metal cations in FOCs was Zn (Fig. S5). The ICP mass measurement of *Tl*Dps1 uncovered the presence of ~ 24 Zn cations per dodecamer other than Fe cations, supporting the Zn‐binding FOCs.

### Novel metal‐binding sites located on the surface of the protein

**Fig. 3:**
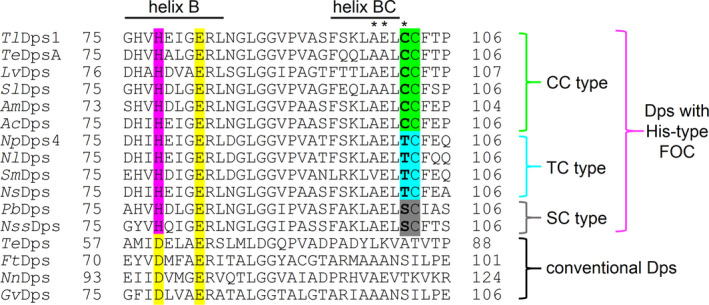
Part of the multiple sequence alignment from the middle of helix B to the end of helix BC for cyanobacterial Dps proteins; Dps proteins from *T. elongatus* BP‐1 (*Te*DpsA, BAC08166; *Te*Dps, BAC10021), *Leptolyngbya valderiana* (*Lv*Dps, WP_063717931), *Synechococcus lividus* (*Sl*Dps, WP_099798363), *Acaryochloris marina* (*Am*Dps, WP_012163802), *Anabaena cylindrica* (*Ac*Dps, WP_015214761), *N. punctiforme* (*Np*Dps4, WP_012412040), *Nostoc linckia* (*Nl*Dps, WP_096541273), *Scytonema millei* (*Sm*Dps, WP_069348791), *Pseudanabaena biceps* (*Pb*Dps, WP_009627941), *Neosynechococcus sphagnicola* (*Nss*Dps, WP_036530773), *Fischerella thermalis* (*Ft*Dps, WP_102178021), *Nodosilinea nodulosa* (*Nn*Dps, WP_017301757), and *Gloeothece verrucosa* (*Gv*Dps, WP_013321962). The highly conserved amino acid residues at the FOCs and His residue of His‐type FOCs are highlighted in yellow and pink, respectively. His‐type FOC‐containing Dps proteins with highly conserved Cys residue (Cys103 in *Tl*Dps1) can be classified into three categories depending on the neighboring amino acid residue in bold; CC (green), TC (blue), and SC (gray) types. Amino acid residues of the OMS1 are indicated by asterisks.

Interestingly, the crystal structure of *Tl*Dps1 clearly featured 12 novel metal‐binding sites located on the outer surface of the protein cage (Fig. 2E). These metal‐binding sites consisted of three amino acid residues (Ala99, Glu100, and Cys102) at the end of the helix BC. There have been several reports of Dps proteins with metal‐binding sites located outside the cavity; however, none has been observed at the end of the helix BC to date [9, 10, 11]. We focused on Cys102 in this novel metal‐binding site because most Dps proteins rarely possess Cys residues, whereas Dps proteins from cyanobacteria usually possess multiple Cys residues. Based on the sequence alignment search, we found that (a) Dps proteins with His‐type FOCs possess highly conserved Cys residues (Cys103 in *Tl*Dps1) and (b) they can be classified into three groups: CC, TC, and SC types (Fig. 3). Most Dps proteins with His‐type FOCs are categorized into the CC type, which consists of Dps proteins possessing continuous two Cys residues at the end of the helix BC (e.g., Cys102 and Cys103 in *Tl*Dps1). The TC type is a class of the Dps proteins possessing one Thr and one Cys residue (e.g., Thr102 and Cys103 in *Np*Dps4); they are found in the order Nostocales; most of these proteins belong to the family Nostocaceae, the filamentous heterocystous cyanobacteria. Conversely, the SC type with one Ser and one Cys residue (e.g., Ser102 and Cys103 in *Pb*Dps) is comparatively rare. In the previously reported *Te*DpsA and *Np*Dps4, metal cations were not observed at these sites, presumably due to the recombinant proteins or the requirement for Ala, Glu, and Cys as metal‐binding amino acid residues. Although the role of the highly conserved Cys residue in Dps proteins with His‐type FOCs (Cys103 in *Tl*Dps1) is currently unclear, the other Cys residue of the CC‐type Dps protein (Cys102 in *Tl*Dps1) could act as a metal‐binding site (outer metal‐binding site 1, OMS1).

To assign the type of metal cations at the OMS1, the apo form of *Tl*Dps1 (*Tl*apoDps1) was prepared by adding a reductant under an anaerobic condition (see the Materials and methods section for details). The UV–vis spectrum of *Tl*apoDps1 displayed a dramatic decrease in absorption bands at ~ 350 nm assignable to the O^2–^‐to‐Fe^3+^ LMCT process (Fig. S3), suggesting that Fe^3+^ ions were successfully reduced and removed from the cavity. SEC‐RALS analysis illustrated that the molecular mass of *Tl*apoDps1 was 232 658, supporting the removal of iron oxyhydroxide clusters. Because the ICP mass measurement of *Tl*apoDps1 disclosed the presence of 24 Zn cations coordinated to FOCs and 12 Fe cations per dodecamer, the type of metal cation coordinated to OMS1 was deduced to be Fe. The anomalous electron density maps obtained from data collected at energies above and below the Zn *K* edge both showed anomalous signals, supporting that the metal cations in OMS1 were not Zn (Fig. S5). To the best of our knowledge, *Tl*Dps1 is the first example with native metal‐binding sites on the surface of the protein among the previously reported Dps proteins.

Furthermore, 12 metal‐binding sites located within pores on the surface of *Tl*Dps1 were also observed in addition to the aforementioned His‐type FOCs and OMS1 (Fig. 2F). These metal‐binding sites consisted of only Asn85 at the end of the helix B (OMS2), indicating the weak interactions between metal cations at OMS2 and the surface of *Tl*Dps1. Oesterhelt and coworkers have reported that the X‐ray crystallographic analysis of iron‐soaked *Hs*DpsA showed the Fe‐binding site at almost the same position of OMS2 in *Tl*Dps1, and they stated about the potential pathway to transport Fe cations through OMS2 pores [8]. According to this report, the type of metal cations in OMS2 was deduced to be Fe, which was supported by the anomalous X‐ray diffraction data (Fig. S5). Interestingly, there have been no reports about translocation sites like OMS2 pores other than ferritin‐like and Dps‐like interfaces along the *C*_3_ symmetry axes in bacterial Dps proteins [34]. Given that the ICP mass measurement of *Tl*apoDps1 indicated the removal of Fe cations at OMS2, pores of OMS2 possibly act as Fe cation channels in *Tl*Dps1.

### Gel electrophoresis analysis of *Tl*Dps1

**Fig. 4:**
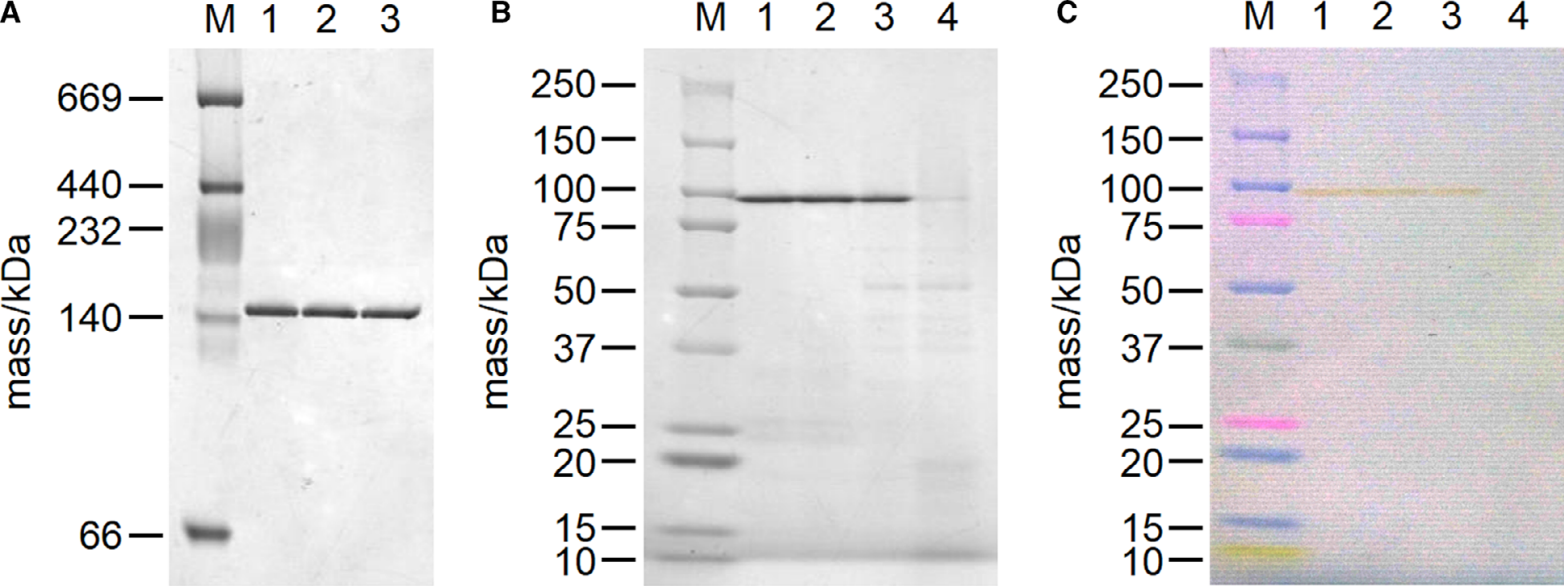
(A) Native PAGE of *Tl*Dps1. Lane 1, *Tl*Dps1 in 10 mm Tris/HCl buffer at pH 8.0; lane 2, *Tl*Dps1 in a buffer solution containing 0.4 m Tris/HCl buffer at pH 7.9, 0.1 m NaCl, 60 mm MgCl_2_, and 10 mm CaCl_2_; lane 3, DNase I‐treated *Tl*Dps1 in a buffer solution containing 0.4 m Tris/HCl buffer at pH 7.9, 0.1 m NaCl, 60 mm MgCl_2_, and 10 mm CaCl_2_. (B) SDS/PAGE of *Tl*Dps1 in 10 mm Tris/HCl buffer at pH 8.0 after incubation for 10 min at 298 (lane 1), 323 (lane 2), 348 (lane 3), and 373 K (lane 4). (C) Color image of the gel for (B) before staining with Coomassie dye. M represents markers.

In native PAGE of *Tl*Dps1, only a yellow 150‐kDa band was observed, supporting the high purity of *Tl*Dps1 (Fig. 4A). The color of the yellow band was caused by the O^2–^‐to‐Fe^3+^ LMCT process, supporting that a yellow 150‐kDa band was assigned to the dodecameric structure containing iron oxyhydroxide clusters even though a smaller molecular mass was suggested by native PAGE (calculated mass of *Tl*Dps1 without metal cations was 248 kDa). A similar result was also observed in *Np*Dps4 [35]. In this report, an ~ 160‐kDa band of the putative dodecamer of *Np*Dps4 (262 kDa) was observed via native PAGE. Concerning SDS/PAGE, a yellow 95‐kDa band was clearly observed when a mixture of the *Tl*Dps1 solution and the sample buffer was incubated for 10 min at 298, 323, or 348 K before loading onto the gel (Fig. 4B). Interestingly, the observed value of 95 kDa was much larger than the expected monomeric molecular mass of ~ 20 kDa. The color of these yellow bands was also caused by the O^2–^‐to‐Fe^3+^ LMCT process (Fig. 4C), suggesting that the dodecameric structure of *Tl*Dps1 was maintained even in the presence of SDS. Although several colorless protein bands assignable to oligomeric structures without iron oxyhydroxide clusters were observed upon increasing the incubation temperature, *Tl*Dps1 potentially possesses high thermostability as expected. In the case of the DpsA protein from *Synechococcus* sp. strain PCC7942, a broad 22‐kDa monomer band was observed when boiling the sample, and a 150‐kDa oligomer band was observed when heating the sample at 343 K before loading onto the gel [36]. This DpsA protein contained single‐stranded DNA that formed a stable Dps–DNA complex. However, the 150‐kDa band of *Tl*Dps1 observed via native PAGE did not change after treatment with DNase (Fig. 4A), suggesting the absence of DNA in *Tl*Dps1. These results strongly supported the high thermostability of *Tl*Dps1 itself.

### Thermostability of *Tl*Dps1

**Fig. 5:**
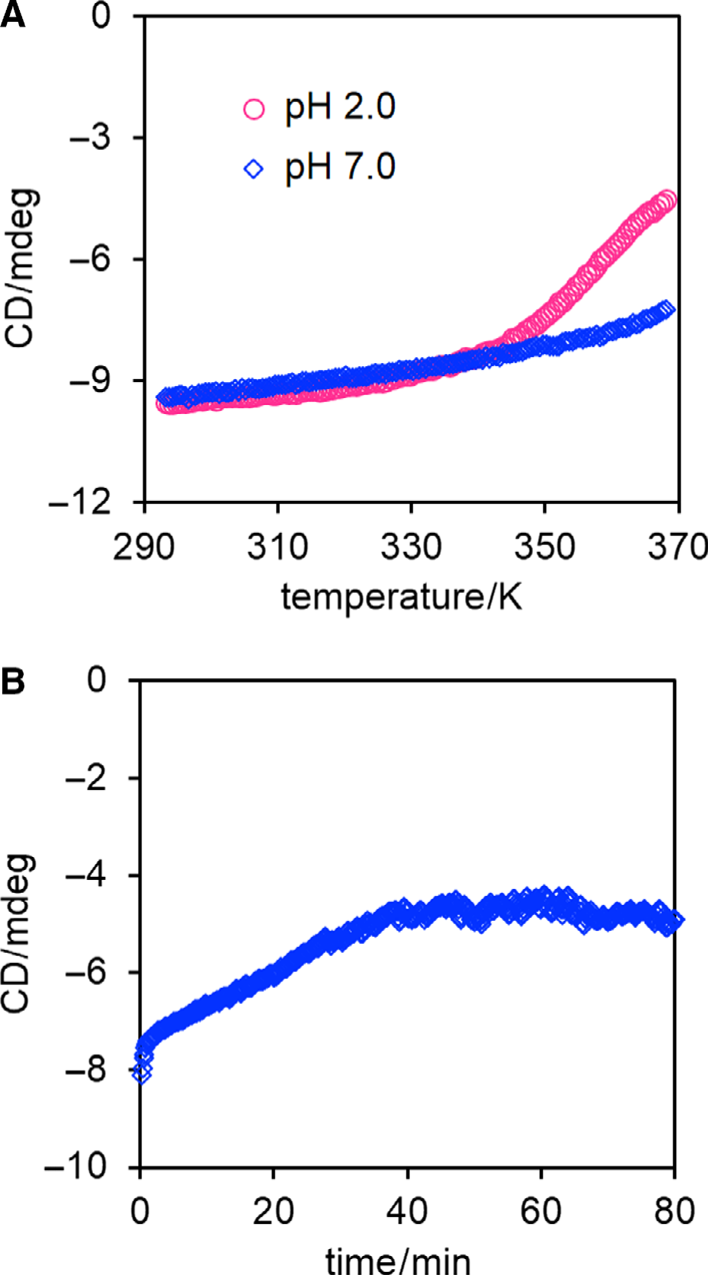
(A) Temperature dependences of CD at 222 nm for *Tl*Dps1 at pH 2 (pink circle) and pH 7 (blue square). (B) Time‐course CD spectrum at 222 nm and 368 K for *Tl*Dps1 at pH 7. Concentrations of the samples were 0.2 mg·mL^−1^.

The temperature dependence of CD at 222 nm was measured to further investigate the thermostability of *Tl*Dps1. However, the transition from the native to the denatured state could not be observed at pH 7.0 owing to the high thermostability of *Tl*Dps1 (Fig. 5A). It has been reported that several thermostable Dps proteins, including *Li*Dps, *Lm*Dps, *Te*Dps, and *Te*DpsA, also do not exhibit the transition at pH 7.0, and thus, their thermal denaturation midpoints (*T*_m_) were determined at pH 2.0 as follows: *T*_m_ = 325 (*Li*Dps), 336 (*Lm*Dps), 345 (*Te*DpsA), and 351 K (*Te*Dps) [6, 12]. Surprisingly, *T*_m_ of *Tl*Dps1 at pH 2.0 could not be determined because the transition was not completed even at 368 K, illustrating the exceedingly high thermostability of *Tl*Dps1 (Fig. 5A). Accordingly, the structural half‐life (*t*_1/2_) of *Tl*Dps1 at pH 7.0 and 368 K was investigated by plotting the CD at 222 nm as a function of time. The CD increased with increasing the time and saturated at ~ 40 min, resulting in a *t*_1/2_ of 14 min (Fig. 5B). Based on the X‐ray crystallographic analysis, multiple inter‐subunit hydrogen bonding networks that stabilized the quaternary structure were observed (Table S2). Most of the residues were well conserved in His‐type FOC‐containing Dps proteins; however, several hydrogen bonds were formed with different amino acid residues compared with the findings for *Te*DpsA or *Np*Dps4, namely Asn85–Phe52, Ser94–Asp109, Gln61–Tyr60, Pro18–Gln126, Ile19–Arg133, Arg148–Glu152, Ile8–Val111, Gln9–Val111, and Ser57–Asp172. Particularly, Gln61 in *Tl*Dps1 formed a new hydrogen bond with Tyr60, which was not observed in *Te*DpsA and *Np*Dps4, partly contributing to the high thermostability. In addition, metal cations coordinated to OMS1 in *Tl*Dps1, presumably enhancing the thermostability. It is noteworthy that *Tl*Dps1 possesses the highest thermostability among all reported Dps proteins.

## Conclusions

In conclusion, a His‐type FOC‐containing Dps protein form *Tl.* O‐77 (*Tl*Dps1) was purified and characterized. X‐ray crystallographic analysis revealed that *Tl*Dps1 possessed Zn‐binding His‐type FOCs together with novel Fe‐binding sites located on the surface of the protein (OMS1), which was discovered for the first time. By focusing on the Cys residue of the OMS1, we found that Dps proteins with His‐type FOCs could be classified into three categories depending on the amino acid (Cys, Thr, or Ser) next to the highly conserved Cys residue (Cys103 in *Tl*Dps1) based on a sequence alignment search. *Tl*Dps1 belongs to the CC type, which possesses two continuous Cys residues at the end of the helix BC, and one of the Cys residues (Cys102) acts as a metal‐binding site. Thus, the metal‐coordinated OMS1 is considered unique to CC‐type Dps proteins in cyanobacteria. In addition, the outer Fe‐binding sites (OMS2) besides OMS1 were also discovered for the first time in bacterial Dps proteins. Considering the weak interactions between Fe cations and the surface of *Tl*Dps1, pores of OMS2 possibly act as Fe cation channels in *Tl*Dps1 as previously reported in a study of *Hs*DpsA. Moreover, *Tl*Dps1 possessed the highest thermostability among all reported Dps proteins. Further investigations of the physiological roles of metal cations at OMS1 would help to clarify the mechanism by which cyanobacterial Dps proteins regulate iron homeostasis and facilitate defense activity against ROS.

## Conflict of interest

The authors declare no conflict of interest.

## Author contributions

TM, KY, and SO devised the study. KY conducted initial isolation experiment. TM conducted experiments, analyzed the data, and wrote the manuscript. TT and YK conducted the crystal structure solution and refinement. All authors revised the manuscript.

## Supporting information


**Table S1**. Crystallographic data of *Tl*Dps1.
**Table S2**. Inter‐subunit hydrogen bonds in *Tl*Dps1.
**Fig. S1** **.** Alignment of amino acid sequences of Dps‐encoding genes in *Tl*. O‐77 and selected crystallographically characterized Dps proteins; Dps proteins from *Thermosynechococcus elongatus* BP‐1 (*Te*Dps, BAC10021; *Te*DpsA, BAC08166), *N. punctiforme* (*Np*Dps4, WP_012412040), *Agrobacterium tumefaciens* (*At*Dps, QCL94627), *Brucella melitensis* (*Bm*Dps, SUW36555), *Microbacterium arborescens* (*Ma*Dps, OAZ39663), *Escherichia coli* (*Ec*Dps, AAD28292), *Yersinia pestis* (*Yp*Dps, SUQ38876), *Streptomyces coelicolor* (*Sc*DpsA, TDZ12299; *Sc*DpsC, TDZ11827), *M. smegmatis* (*Ms*Dps1, STZ34952; *Ms*Dps2, SUA34587), *L. innocua* (*Li*Dps, SPX75031), *Listeria monocytogenes* (*Lm*Dps, RLQ56992), *Vibrio cholerae* (*Vc*Dps, SNC54720), *Bacillus brevis* (*Bb*Dps), *Kineococcus radiotolerans* (*Kr*Dps, WP_012085961), *D. radiodurans* (*Dr*Dps1, WP_010888891; *Dr*Dps2, WP_010883959), and *Halobacterium salinarum* (*Hs*DpsA, WP_010903826). The highly conserved residues at the FOC centers and His residues of His‐type FOC centers are highlighted in yellow and pink, respectively. Characteristic amino acid residues of CC‐ and TC‐type Dps proteins are highlighted in green and blue, respectively. The amino acid residues in bold for *Tl*Dps1 represent the amino acid sequence observed and assigned by the ISD‐MALDI‐TOF mass measurement.
**Fig. S2** **.** Image of single crystals of *Tl*Dps1.
**Fig. S3** **.** UV–vis spectra of *Tl*Dps1 (solid line) and *Tl*apoDps1 (dashed line) in 10 mm Tris/HCl buffer at pH 8.0. Concentrations of the samples were 0.1 mg·mL^−1^.
**Fig. S4** **.** CD spectra of *Tl*Dps1 in 50 mm sodium phosphate buffer at pH 7.0 (blue line) and in HCl aqueous solution at pH 2.0 (pink line). Concentrations of the samples were 0.2 mg·mL^−1^.
**Fig. S5** **.** Cartoon representations of FOC (A, B), OMS1 (C, D), and OMS2 (E, F) in *Tl*Dps1 based on the X‐ray crystallographic analysis. 1.25‐Å (red; A, C, E) and 1.32‐Å (green; B, D, F) wavelength anomalous electron density maps contoured at 2.3σ are superimposed on metal cations. Zn and Fe atoms are represented as gray and orange spheres, respectively.

## Data Availability

The refined structure of *Tl*Dps1 has been deposited in the RCSB Protein Data Bank under accession code 6LKP.
