# Supplementary material for: Biochemical and structural characterization of a thermostable Dps protein with His‐type ferroxidase centers and outer metal‐binding sites
Source: FEBS Open Bio. 2020 May 28;10(7):1219–29. doi: 10.1002/2211-5463.12837 (PMC7327923; doi:10.1002/2211-5463.12837)
Supplement: Supplementary file 1 — Table S1. Crystallographic data of TlDps1. Table S2. Inter‐subunit hydrogen bonds in TlDps1. Fig. S1 . Alignment of amino acid sequences of Dps‐encoding genes in Tl. O‐77 and selected crystallographically characterized Dps proteins; Dps proteins from Thermosynechococcus elongatus BP‐1 (TeDps, BAC10021; TeDpsA, BAC08166), N. punctiforme (NpDps4, WP_012412040), Agrobacterium tumefaciens (AtDps, QCL94627), Brucella melitensis (BmDps, SUW36555), Microbacterium arborescens (MaDps, OAZ39663), Escherichia coli (EcDps, AAD28292), Yersinia pestis (YpDps, SUQ38876), Streptomyces coelicolor (ScDpsA, TDZ12299; ScDpsC, TDZ11827), M. smegmatis (MsDps1, STZ34952; MsDps2, SUA34587), L. innocua (LiDps, SPX75031), Listeria monocytogenes (LmDps, RLQ56992), Vibrio cholerae (VcDps, SNC54720), Bacillus brevis (BbDps), Kineococcus radiotolerans (KrDps, WP_012085961), D. radiodurans (DrDps1, WP_010888891; DrDps2, WP_010883959), and Halobacterium salinarum (HsDpsA, WP_010903826). The highly conserved residues at the FOC centers and His residues of His‐type FOC centers are highlighted in yellow and pink, respectively. Characteristic amino acid residues of CC‐ and TC‐type Dps proteins are highlighted in green and blue, respectively. The amino acid residues in bold for TlDps1 represent the amino acid sequence observed and assigned by the ISD‐MALDI‐TOF mass measurement. Fig. S2 . Image of single crystals of TlDps1. Fig. S3 . UV–vis spectra of TlDps1 (solid line) and TlapoDps1 (dashed line) in 10 mm Tris/HCl buffer at pH 8.0. Concentrations of the samples were 0.1 mg·mL−1. Fig. S4 . CD spectra of TlDps1 in 50 mm sodium phosphate buffer at pH 7.0 (blue line) and in HCl aqueous solution at pH 2.0 (pink line). Concentrations of the samples were 0.2 mg·mL−1. Fig. S5 . Cartoon representations of FOC (A, B), OMS1 (C, D), and OMS2 (E, F) in TlDps1 based on the X‐ray crystallographic analysis. 1.25‐Å (red; A, C, E) and 1.32‐Å (green; B, D, F) wavelength anomalous electron density maps contoured at 2.3σ [file FEB4-10-1219-s001.pdf]

Supporting Information for

Biochemical and structural characterization of a thermostable Dps protein with His-type ferroxidase centers and outer metal-binding sites

Takuo Minato[1,2]*, Takamasa Teramoto[3], Yoshimitsu Kakuta[3,4], Seiji Ogo[1,2,5], Ki-Seok Yoon[1,2,5]*

1 Department of Chemistry and Biochemistry, Graduate School of Engineering, Kyushu University, 744 Moto-oka, Nishi-ku, Fukuoka 819-0395, Japan

2 International Institute for Carbon-Neutral Energy Research (WPI-I2CNER), Kyushu University, 744 Moto-oka, Nishi-ku, Fukuoka 819-0395, Japan

3 Department of Bioscience and Biotechnology, Faculty of Agriculture, Kyushu University, 744 Moto-oka, Nishi-ku, Fukuoka 819-0395, Japan

4 Laboratory of Structural Biology, Graduate School of System Life Sciences, Kyushu University, 744 Moto-oka, Nishi-ku, Fukuoka 819-0395, Japan

5 Center for Small Molecule Energy, Kyushu University, 744 Moto-oka, Nishi-ku, Fukuoka 819-0395, Japan

Table S1. Crystallographic data of *Tl*Dps1.

**Data Collection**	
Wavelength (Å)	1.25
Space group	$P4_322$ (No. 95)
Cell Dimensions	
a, b, c (Å)	92.8, 92.8, 261.1
α, β, γ (°)	90.0, 90.0, 90.0
Resolution (Å)	46.39–2.90 (3.00–2.90)*
R-merge	0.128 (1.606)
R-pim	0.035 (0.455)
$CC_{1/2}$	0.999 (0.627)*
$I/\sigma(I)$	12.1 (1.2)*
Completeness (%)	100 (100)*
Redundancy	14.0 (13.3)*
**Refinement**	
Resolution (Å)	46.39-2.9
No. reflections	48249
R_{work} /R_{free} (%)	25.3/31.5
No. of Atoms	
Protein	7968
Metal	24
B-Factors	
Protein	95.4
Metal	140.9
RMS Deviations	
Bond lengths (Å)	0.003
Bond angles (°)	0.625
Ramachandran plot (%)	
Favored	95.44
Allowed	4.56
PDB ID	6LKP

*Values in parentheses are for the highest resolution shell.

Table S2. Inter-subunit hydrogen bonds in *Tl*Dps1.

dimeric interface
Q41–Q48
Y70–H78
I8–V111
Q9–V111
N85–F52
S94–D109
trimeric Dps-like interface
E58–S173
E58–L174
S57–D172
Q61–Y60
Q61–Q61
G56–H167
trimeric ferritin-like interface
Q126–P18
R133–I19
E152–R148
E159–R83
H167–N85

```
                                                                                   helix A
TlDps1    1   -------------------------------------------------MAITASPIQTFEQMKDNPIGLEMNVTTAVCEGFNI   35
TlDps2    1   --------------------------------------------------MTTETLVRAFGQVGPNPVGFDLDITTAICEGLNL   34
TlDps3    1   ------------------------------------------------------------------MTTTLEARQTALVKALNR   18
TlDps4    1   ------------------------------------------------MGSNGRSTNGKSQPLYATRIDLSSEIRTKVIAILNH   36
NpDps4    1   -------------------------------------------------MSETQTLLRNFGNVYDNPVLLDRSVTAPVTEGFNV   35
TeDpsA    1   -------------------------------------------------MTTSALPRQAFGEMADTVILLEKATTTPICEGMNR   35
TeDps     1   -------------------------------------------------------------------MSATTTLKEQVLTTLKR   17
AtDps     1   ------------------------------------------------------------MKTHKTKNDLPSNAKSTVIGILNE   24
BmDps     1   ---------------------------------------------------------MPKKSMHATRNDLPSNTKTTMIALLNE   27
MaDps     1   ---------------------------------------------------------MTDTNITTPALTADPEVAAAAAQFLTP   27
EcDps     1   -------------------------------------------------MSTAKLVKSKATNLLYTRNDVSDSEKKATVELLNR   35
YpDps     1   -------------------------------------------------MSTAKLVKTKPSELLYTRNDVEEHVKVATIKRLNQ   35
ScDpsA    1   --------------------------------------------------------MTHDLTPKYTVPGIEREAAGRLIGVLRL   28
ScDpsC    1   ---------------------------MSSPKPKPPSSAEHRSDGSQPWLHQKGRTIQEFGTVKQFPVALTMDTRLYSCQRLNK   57
MsDps1    1   -------------------------------------------------------------MTSFTIPGLSDKKASDVADLLQK   23
MsDps2    1   -----------------------------------------------------------MSARRTESDIQGFHATPEFGGNLQK   25
LiDps     1   ---------------------------------------------------------------------MKTINSVDTKEFLNH   15
LmDps     1   ---------------------------------------------------------------------MKTINSVDTKEFLNH   15
VcDps     1   --------------------------------------------------------------MATNLIGLDTTQSQKLANALNN   22
BbDps     1   ---------------------------------------------------------------------MKTSIQQLVAVLLNR   15
KrDps     1   ----------------------------------------------------MTTIHDVQTTGLTQDAVTGFDASSRLNAGLQE   32
DrDps1    1   -----------------MTKKSTKSEAASKTKKSGVPETGAQGVRAGGADHADAAHLGTVNNALVNHHYLEEKEFQTVAETLQR   67
DrDps2    1   MRHSVKTVVVVSSLLLGTALAGGAGAQSAGNGVPSTNVNTPAPNTGQSTAQNTNTASPLPYNRATTLPAAGTEDLKKSVQALQN   84
HsDpsA    1   ------------------------------------------------MSTQKNARATAGEVEGSDALRMDADRAEQCVDALNA   36

                  helix A                   helix B                    helix BC        helix C
TlDps1   36   VLASFQALYLQYQKHHFVVEGSEFYQLHEFFSESYDEVQGHVHEIGERLNGLGGVPVASFSKLAELCCFTPEPDGVFSCRAMVE  119
TlDps2   35   AYASFQALYLQYQKHHFVVEGAEFYSIHEFFQESYNATQSHAHDLAERLNGLGGIPAGSFATLSDLCCFAPEPDGAYTCRAMLE  118
TlDps3   19   EQANTLVAYLNYKKYHWMTYGPLFRDLHLLFEEHGNEIFAMLDELAERSLMLDGTPIADPADYLPAATVKPSK-GKLKVREMVE  102
TlDps4   37   SLASTLDLKTQVKQAHWNVKGLQFYQLHELFDEMASELEEYVDMVAERVTALGGLAVGTARTAAAQSILPEYPFDILDGKDHVI  120
NpDps4   36   VLASFQALYLQYQKHHFVVEGSEFYSLHEFFNESYNQVQDHIHEIGERLDGLGGVPVATFSKLAELTCFEQESEGVYSSRQMVE  119
TeDpsA   36   LLASFQALYLQYQKHHFVVEGAEFYPLHQFFQDCYEQVQDHVHALGERLNGLGGVPVAGFQQLAALCCFTPEPEGAFNCRQMLS  119
TeDps    18   EQANAVVMYLNYKKYHWLTYGPLFRDLHLLFEEQGSEVFAMIDELAERSLMLDGQPVADPADYLKVATVTPSS-GQLTVKQMIE  101
AtDps    25   SLASVIDLALITKQAHWNLKGPQFIAVHELLDTFRTQLDNHGDTIAERVVQLGGTALGSLQSVSSTTKLKAYPTDIYKIHDHLD  108
BmDps    28   NLAATIDLALITKQAHWNLKGPQFIAVHEMLDGFRAELDDHVDTIAERAVQIGGTAYGTTQVVVKESRLKPYPTDIYAVHDHLV  111
MaDps    28   VVHKMQALVVNGKQAHWNVRGSNFIAIHELLDSVVAHAQDYADTAAERIVALGLPIDSRVSTMADKTSTAV-PAGFAQWQDEIK  110
EcDps    36   QVIQFIDLSLITKQAHWNMRGANFIAVHEMLDGFRTALIDHLDTMAERAVQLGGVALGTTQVINSKTPLKSYPLDIHNVQDHLK  119
YpDps    36   MVIQFIDLSLITKQAHWNMRGANFVAVHEMLDGFRTALTDHLDTFAERAVQLGGVALGTAQVINDKTPLKSYPTNIHSVQEHLK  119
ScDpsA   29   RLHALNDLHLTLKHVHWNVVGPHFIAVHEMIDPQVDQVRDMADDVAERIAALGGVAQGTPGALVAERKWDDYSIGRADAIAHLG  112
ScDpsC   58   VLADTRILHDLYKKYHWLMRGATFYQLHLLLDKHAGEQLELIDTVAERVQTLGGVAVGDPRHVAEITTVPRPPDGVEEVPSMLS  141
MsDps1   24   QLSTYNDLHLTLKHVHWNVVGPNFIGVHEMIDPQVELVRGYADEVAERIATLGKSPKGTPGAIIKDRTWDDYSVERDTVQAHLA  107
MsDps2   26   VLVDLIELSLQGKQAHWNVVGSNFRDLHLQLDELVDFAREGSDTIAERMRALDAVPDGRSDTVAATTTLPEFPAFERSTADVVD  109
LiDps    16   QVANLNVFTVKIHQIHWYMRGHNFFTLHEKMDDLYSEFGEQMDEVAERLLAIGGSPFSTLKEFLENASVEEAP---YTKPKTMD   99
LmDps    16   QVANLNVFTVKIHQIHWYMRGHNFFTLHEKMDDLYSEFGEQMDEVAERLLAIGGSPFSTLKEFLENASVEEAP---YTKPKTMD   99
VcDps    23   LLANYQVFYMNTRGYHWNIQGKEFFELHAKFEEIYTDLQLKIDELAERILTLSARPMHSFSGYLKAAQIKEHT-DSIDGRSSMQ  106
BbDps    16   QVANWVVLYVKLHNFHWNVNGPNFFTLHEKFEELYTEASGHIDTLAERVLSIGGSPIATLAASLEEASIKEAT-GGESAAEMVS   99
KrDps    33   VLVDLTALHLQGKQAHWNIVGENWRDLHLQLDTLVEAARGFSDDVAERMRAVGGVPDARPQTVAA-SRIGDVGPDEIDTRACVE  115
DrDps1   68   NLATTISLYLKFKKYHWDIRGRFFRDLHLAYDEFIAEIFPSIDEQAERLVALGGSPLAAPADLARYSTVQVPQETVRDARTQVA  151
DrDps2   85   TLTELQALQLQTKQAHWNVSGTLWYTLHELLQDHYEGISKFADDVAERQLSVGASSDGRAITIVAASRLPEIPGGFLDDAQVIQ  168
HsDpsA   37   DLANVYVLYHQLKKHHWNVEGAEFRDLHLFLGEAAETAEEVADELAERVQALGGVPHASPETLQAEASVDVEDEDVYDIRTSLA  120

                     helix C                   helix D
TlDps1  120   HDLSAE----QEIIKVIRRQAGQAESLGDRATRHLY-EKILLESEDRAFHLSHFLAHDSLTPAFTLASQN--------------  184
TlDps2  119   HDLAAE----QAAIDLLRRLSVQAESLGDRATRYLY-DEILLKTEDRAFHINHFLAHDSLTPAFMTA-----------------  180
TlDps3  103   EAIATH----EHIITEMHEDAELADDAGDIGTADLL-TRLVQVHQKHRWFLKEILKQGDGLLDG---------------------  160
TlDps4  121   ALAERY----AAYGKLVRESIDATDDLGDADTADLY-TEISRDIDKRLWFLEAHLQTAVEEVVSATAASEVAEPKAKKTAKGAK  199
NpDps4  120   NDLAAE----QAIIGVIRRQAAQAESLGDRGTRYLY-EKILLKTEERAYHLSHFLAKDSLTLGFVQAAQS--------------  184
TeDpsA  120   NDLQAE----QAIIGVLRQQATQAESLGDRATAYLY-DQILLKTEERAYHIGHFLANDSLKV----------------------  176
TeDps   102   EAIANH----ELIITEMHQDAEIATEAGDIGTADLY-TRLVQTHQKHRWFLKEFLAKGDGLVS----------------------  158
AtDps   109   ELIERY----GEVANMIRKAIDDSDEAGDPTTADIF-TAASRDLDKSLWFLEAHVQEKS-------------------------  162
BmDps   112   ALIERY----GDVANLVRKSIKDADDAGDDDTADIF-TAASRSLDKALWFLEAHVQESN-------------------------  165
MaDps   111   AIVSDI----DAALVDLQAAIDGLDEV-DLSSQDVA-IEIKRGVDKDRWFLLAHLAE---------------------------  161
EcDps   120   ELADRY----AIVANDVRKAIG---EAKDDDTADIL-TAASRDLDKFLWFIESNIE----------------------------  167
YpDps   120   ALAERY----AIVANDIRKAIT---EVEDENSADMF-TAASRDLDKFLWFIESNIE----------------------------  167
ScDpsA  113   ALDVVY----TGVVEGMRAAVEEAGKI-DPATEDLL-IGQLRDLEQFQWFVRAHLESAGGALATGSATSETEAAARGAEVA---  187
ScDpsC  142   RLLEAH----ELILTECHDAAARTQEYGDDGTNDLLVSEVLRTNELQAWFVAEHLVDTPLVHA---------------------  200
MsDps1  108   ALDLVY----NGVIEDTRKSIEKLEDL-DLVSQDLL-IAHAGELEKFQWFVRAHLESAGGQLTHEGQSTEKGAADKARRKSA--  183
MsDps2  110   LITTRI----NATVDTIRRVHDAV-DAEDPSTADLL-HGLIDGLEKQAWLIRSENRKV--------------------------  161
LiDps   100   QLMEDLVGTLELLRDEYKQGIELTDKEGDDVTNDML-IAFKASIDKHIWMFKAFLGKAPLE-----------------------  156
LmDps   100   QLMEDLVGTLELLRDEYQQGIELTDKEGDNVTNDML-IAFKASIDKHIWMFKAFLGKAPLE-----------------------  156
VcDps   107   GLVDGF----SILLHQQRDILELAGETGDEGTSALM-SDYIREQEKLVWMLNAWLK-----------------------------  156
BbDps   100   SVVNDF----VDLVGELKVARDVADEADDEATADML-DAIEAGLEKHVWMLEAFLE-----------------------------  149
KrDps   116   AIVALV----RHTVDTIRRVHDPI-DAEDPASADLL-HAITLELEKQAWMIGSENRSPRRR-----------------------  170
DrDps1  152   DLVQDL----SRVGKGYRDDSQACDEANDPVTADMY-NGYAATIDKIRWMLQAIMDDERLD-----------------------  207
DrDps2  169   FFTYQY----ETVGQRIHQRVGDVEKV-DPTTANLL-QEVEHIIEKYQWQMRAFLQNTPTDPNTGFDINNGKPVPLRGR-----  241
HsDpsA  121   NDMAIY----GDIIEATREHTELAENLGDHATAHML-REGLIELEDDAHHIEHYLEDDTLVTQGALE-----------------  182
```

Fig. S1. Alignment of amino acid sequences of Dps encoding genes in *Tl*. O-77 and selected crystallographically characterized Dps proteins; Dps proteins from *Thermosynechococcus elongatus* BP-1 (*Te*Dps, BAC10021; *Te*DpsA, BAC08166), *Nostoc punctiforme* (*Np*Dps4, WP_012412040), *Agrobacterium tumefaciens* (*At*Dps, QCL94627), *Brucella*

melitensis (*Bm*Dps, SUW36555), *Microbacterium arborescens* (*Ma*Dps, OAZ39663), *Escherichia coli* (*Ec*Dps, AAD28292), *Yersinia pestis* (*Yp*Dps, SUQ38876), *Streptomyces coelicolor* (*Sc*DpsA, TDZ12299; *Sc*DpsC, TDZ11827), *Mycobacterium smegmatis* (*Ms*Dps1, STZ34952; *Ms*Dps2, SUA34587), *Listeria innocua* (*Li*Dps, SPX75031), *Listeria monocytogenes* (*Lm*Dps, RLQ56992), *Vibrio cholerae* (*Vc*Dps, SNC54720), *Bacillus brevis* (*Bb*Dps), *Kineococcus radiotolerans* (*Kr*Dps, WP_012085961), *Deinococcus radiodurans* (*Dr*Dps1, WP_010888891; *Dr*Dps2, WP_010883959), and *Halobacterium salinarum* (*Hs*DpsA, WP_010903826). The highly conserved residues at the FOC centers and His residues of His-type FOC centers are highlighted in yellow and pink, respectively. Characteristic amino acid residues of CC- and TC-type Dps proteins are highlighted in green and blue, respectively. The amino acid residues in bold for *Tl*Dps1 represents the amino acid sequence observed and assigned by the ISD-MALDI-TOF mass measurement.

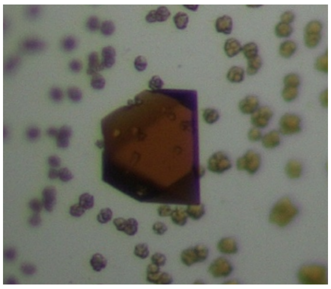

Fig. S2. Image of single crystals of *Tl*Dps1.

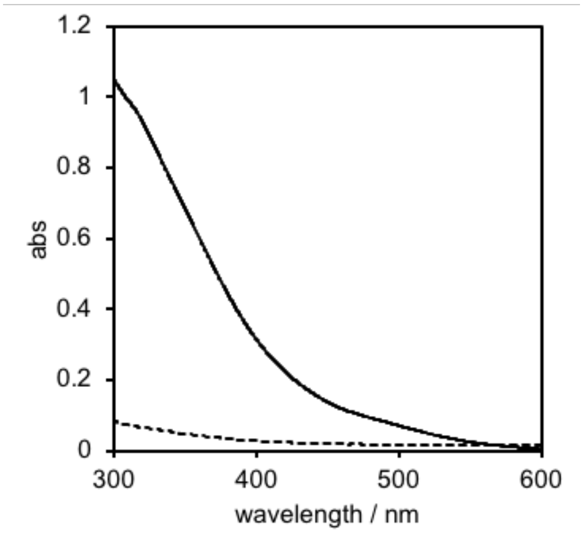

Fig. S3. UV-vis spectra of *Tl*Dps1 (solid line) and *Tl*apoDps1 (dashed line) in 10 mM Tris-HCl buffer at pH 8.0. Concentrations of the samples were 0.1 mg/mL.

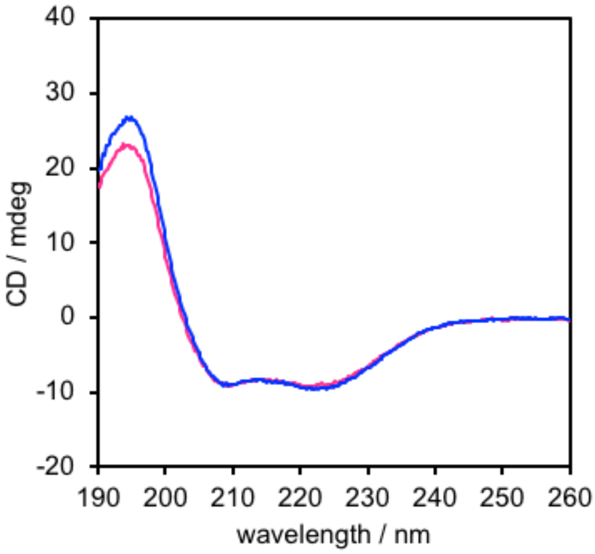

Fig. S4. CD spectra of *Tl*Dps1 in 50 mM sodium phosphate buffer at pH 7.0 (blue line) and in HCl aqueous solution at pH 2.0 (pink line). Concentrations of the samples were 0.2 mg/mL.

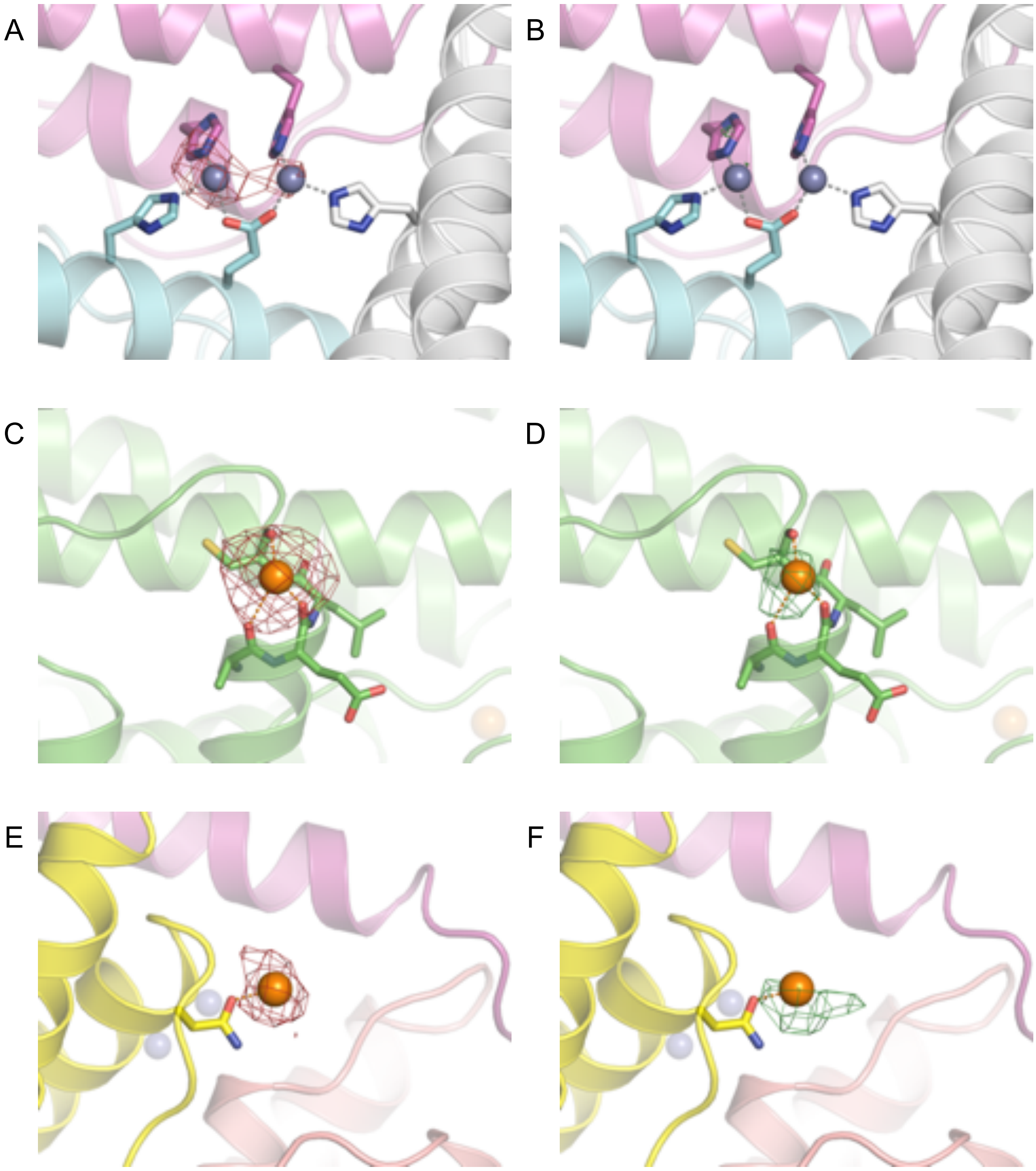

Fig. S5. Cartoon representations of FOC (A, B), OMS1 (C, D), and OMS2 (E, F) in *Tl*Dps1 based on the X-ray crystallographic analysis. 1.25-Å (red; A, C, E) and 1.32-Å (green; B, D, F) wavelength anomalous electron density maps contoured at 2.3σ are superimposed on metal cations. Zn and Fe atoms are represented as gray and orange spheres, respectively.
